# Poisoning by Strychnine—Case of William Palmer

**Published:** 1856-10

**Authors:** J. C. Morfett

**Affiliations:** Chicago


### ARTICLE III.—*Poisoning by Strychnine—Case of Wm. Palmer.* By J. C. Morfett, M.D., Chicago.

The evidence in the celebrated case of William Palmer, tried and convicted of poisoning John Parsons Cook with Strychnine, is of so much interest, both in a medico-legal point of view and on account of the high position of the medical witnesses, that we will give a synopsis of it.

Mr. Cook was a sporting gentleman, of Rugely, England, intimate with Palmer, and was to have received £2,000 on Monday, Nov. 19th, 1855, in consequence of his horse having won a race, Nov. 13th. On Wednesday, Nov. 14th, the day after the races, Palmer and Cook were drinking brandy and water. After drinking part of a glass, Cook said, "There's something in it, it burns my throat. Palmer took up the glass and, drinking the balance, said, "There's nothing in it." Cook, after that, was seized with such violent vomiting that he was obliged to go to bed, but was relieved in the night. The next day Cook went to the races. On Friday, 16th, he dined with Palmer, and went to bed that night perfectly well. On Saturday, 17th, at an early hour, Palmer came to Cook at the "Talbot Arms," and ordered coffee for him. Palmer gave Cook the coffee, and, immediately after taking it, the same symptoms set in which had occurred to him on Wednesday. He continued sick all of that and the next day. On Monday, Palmer, who had been much of the time with Cook, left for London, and immediately Cook began to improve—sat up for several hours, and was altogether much better. Palmer returned to Rugely that same night, Monday 19th, at 9 o'clock, and at once visited Cook at the "Talbot Arms," and from that time until 10 or 11 o'clock was continually in and out of Cook's room. In the course of the evening he went to an apothecary, and bought three grains of strychnine. Mr. Bamford, the medical attendant, had that day sent to Cook some pills, such as he had previously prescribed, containing half a grain of calomel, half a grain of morphine, and four grains rhubarb. Palmer generally came in to administer the medicine. There is no doubt Cook took pills on Monday night: when Palmer left Cook that night, he was very comfortable, but, at 11, the servants of the house were alarmed by cries from Cook's room, and they found him in great agony and intense pain, shouting murder. *He was flinging his arms wildly about, and his whole body was convulsed. He was perfectly conscious*, and desired Palmer to be sent for. Palmer came directly and administered some medicine to Cook, which he vomited almost immediately. Shortly he became more calm, and called upon the servants to rub his limbs. He gradually fell into repose, and began to dose.

The next morning, Tuesday 20th, Cook was comparatively comfortable, and he was ordered to take pills at bedtime. These pills were made by Mr. Bamford, at his surgery, but were given to Palmer to administer to Cook. The latter vomited as soon as he took them. That night he was attacked as before, screaming violently, gasping for breath; his body convulsed with cramps and spasms, and his neck rigid. Cook asked Palmer for the same medicine which had relieved him before, and Palmer brought two pills, which he told the medical attendant were of ammonia. The sick man swallowed these pills, but brought them up immediately. He was instantly seized with violent convulsions; by degrees his body began to stiffen out, then suffocation commenced. He entreated to be raised, but his body was "stiff as iron," and it could not be done. He then said, Pray turn me over," and they did turn him over, when he gasped for breath and in a few moments died. Previously Cook had suffered from syphilis, but had been cured for some time.

At the coroner's inquest, Mr. Bamford, the medical attendant, testified that he died of apoplexy. At the post mortem examination, which took place Nov. 26th, six days after death, the body was found stiff and the hands clenched. The abominal viscera and brain were quite healthy, and normal in every respect; the heart was contracted, and contained no blood; the blood was in a fluid state; the spinal cord was not minutely examined, but found, as far as examined, in a healthy state; the stomach was opened its whole length, and with its contents and the intestines put in again for analysis. On the 25th January, two months after death, the body was examined again, and with the same result, except that on the spinal cord were found certain granules. This, Dr. Monckton said, he had frequently seen in old people, but was not sufficient to account for the symptoms.

Mr. B. Curling, who was examined, testified that he was familiar with tetanus, having written a work on the subject, and did not think the symptoms of Cook referable to that disease, as the sudden onset of the symptoms and their rapid subsidence are not consistent with either of its two forms, viz., idiopathic or traumatic.

Sir. B. Brodie, judging from the symptoms which accompanied Mr. Cook's death, was of opinion that, so far as there was a general contraction of the muscles, they resembled those of traumatic tetanus; but, as to the course these symptoms took, they were entirely different. He did not think death, in this case, arose from what is ordinarily called tetanus, either idiopathic or traumatic; neither were they the result of apoplexy or epilepsy.

Mr. Solly's testimony was substantially the same.

Dr. Alfred S. Taylor testified as follows:—I made a careful analysis of the stomach and intestines, without finding any trace of strychnia. I found antimony in the liver, one kidney, and spleen. I gave it as my opinion that he had died from the administration of that substance, because I was told that he had been taken suddenly ill, and died in convulsions. I did not then know his symptoms, but now think they were referable to strychnine. I have made experiments with rabbits; and, in two instances where strychnine was administered, have been unable to detect it after death. The symptoms of poisoning by strychnine are nearly uniform. In about five or six minutes, when the poison begins to act, there is a trembling, quivering motion of the whole muscles of the body; there is then a sudden paroxysm or fit, the fore-legs and hind-legs are stretched out, the head and tail are drawn back in the form of a bow, jaws spasmodically closed, and eyes prominent. After a short time there is a slight remission of the symptoms, and the animal appears to lie quiet, but the slightest noise or touch produces another paroxysm. Sometimes there is a sort of shriek as if the animal suffered pain; the heart beats violently after a fit, and, after a succession of fits, the animal dies quietly. Sometimes the animal dies during a paroxysm, and I only know that death has occurred, from holding my hand over the heart. The appearances after death differ: In some instances, rigidity continues; in one case the muscles were so strongly contracted for a week that it was possible to hold the body out horizontally by the hind-legs; in some instances I found congestion of the membranes of the spinal cord, but in others there was no departure from the ordinary state of spinal cord and brain: in all cases the heart has been congested, especially the right side. I believe the symptoms in Cook's case to have been produced by strychnine. Strychnine is absorbed, and where only a small quantity has been given at a time, and it has been carried into the circulation, it is impossible to discover it in the tissues.

Dr. G. G. Rees assisted Dr. Taylor in the analysis, and coincided with him entirely in the testimony.

Dr. Christison had also been unable to detect strychnia in the dead body of animals poisoned with it; and agreed with Dr. Taylor that it was sometimes impossible to detect it.

Dr. Thomas Nunnely deposed, that he had always found traces of Strychnia in the body in various stages of decomposition. It could not be absorbed without its presence being detected in the tissues or blood. No amount of putrefaction, within ordinary limits, will prevent the discovery of it—he had found it at the end of forty days. The sudden accession of the symptoms, screaming, vomiting; power to talk, swallow, and move freely, led him to believe that this was not a case of death from that poison. He never knew an animal to scream voluntarily after having taken strychnine. When there is so much spasm of the heart, there must be inability to vomit. He thought Cook's symptoms due to great excitement. In poisoning by strychnine, the rigor mortis is not greater than in death from other causes.

Mr. William Herapath testified, that in all cases in which strychnine had been given, he had been able to find it after death. He has found it in the blood, heart, and urine, besides the stomach. He could detect the fifty-thousandth part of a grain, if unmixed with organic matter.

Dr. Henry Letheby has witnessed many cases of animals poisoned with strychnine, and does not think the symptoms accorded with those attending the death of Cook. Strychnine is the most easily detected of all poisons; never failed to find it when it had been administered; always found the right side of the heart full.

Dr. Ungletson, a pupil of Liebig, testified that he has discovered strychnine in the tissues, urine, blood, and viscera. He administered half a grain to a cat, and detected it in the urine. If a man were poisoned with strychnine, he would expect to find it in the stomach within five or six days—should be confident of finding it even when administered in minute quantities. If a long interval had elapsed, would expect to find it in the liver, spleen, and kidneys.

The evidence in this case was so voluminous, that we have only given an abstract of it on some particular points. It will be seen that there was a conflict of opinion among some of the most eminent medical men who were brought as witnesses; and it was to give the views of these that we undertook this abstract. Those who wish to read the testimony in full, will find it in the *London Lancet*, for July, 1856.

